# Signatures in in vitro infection of NSC-34 mouse neurons and their cell nucleus with *Rickettsia helvetica*

**DOI:** 10.1186/s12866-023-02859-0

**Published:** 2023-04-21

**Authors:** Lena Kask, Carl Påhlson, Karin Staxäng, Kenneth Nilsson

**Affiliations:** 1 Department of Medical Sciences, Section of Clinical Microbiology, Uppsala University, Uppsala, Sweden; 2 Department of Immunology, Genetics and Pathology-Biovis Platform, Uppsala University, Uppsala, Sweden; 3 Department of Medical Sciences, Section of Infectious Diseases, Uppsala University, Uppsala, Sweden

**Keywords:** *Rickettsia helvetica*, Neuron cell line, NSC-34, Actin tail, TEM, qPCR, Western blot, Immunofluorescence

## Abstract

**Background:**

*Rickettsia helvetica*, a spotted fever rickettsia, is transmitted to humans via ticks in Europe, North Africa, and Asia. The central nervous system is a crucial target for rickettsial diseases, which has been reported for 12 of the 31 species, of which *R. helvetica* is one. This study aimed, in an experimental model, to identify characteristics of *R. helvetica* infection in a mouse neuronal cell line, NSC-34.

**Results:**

NSC-34, a fusion cell line of mouse motor spinal cord neurons and neuroblastoma cells, was used as a model. Propagation of *R. helvetica* in neurons was confirmed. Short actin tails were shown at the polar end of the bacteria, which makes it likely that they can move intracellularly, and even spread between cells. Another protein, Sca4, which with the cell adhesion protein vinculin enables the passage of the cell membrane, was expressed during infection. No significant increase in TNFα levels was seen in the infected neurons, which is of interest because TNFα protects the host cell from infection-induced apoptotic death which is crucial for host cell survival. The bacteria were also shown to invade and grow in the cell nucleus of the neuron.

**Conclusions:**

The findings suggest that a *R. helvetica* infection may be harmful to NSC-34 neurons under these in vitro conditions, but the full effects of the infection on the cell need to be studied further, also on human neurons, to also understand the possible significance of this infection in relation to pathogenetic mechanisms.

**Supplementary Information:**

The online version contains supplementary material available at 10.1186/s12866-023-02859-0.

## Background

*Rickettsia* are strictly intracellular bacteria, requiring a vector such as ticks, fleas, or mites for host transmission. They are globally distributed and belong to the family *Rickettsiaceae* comprising two genera, *Rickettsia* and *Orientia* [1, 2]. The genus *Rickettsia*, comprising 31 species, was traditionally categorized into two main groups, the spotted fever group (SFG) and the typhus group (TG), of which two species, *Rickettsia typhi* and *Rickettsia prowazekii*, make up the TG [3]. Spotted fever rickettsioses, on the other hand, are associated with 19 defined species shown to cause human disease, with a clinical spectrum ranging from febrile flu-like illnesses to more severe, even fatal diseases [2, 4].

Moreover, the central nervous system (CNS) is a crucial target of rickettsial diseases, and encephalitis, meningitis as well as facial palsy, or cerebral infarction are known to be caused by twelve of the species [5–10]. Reported evidence suggests that the initial targets for rickettsiae, at the tick feeding site, are the monocytes, which spread the infection further, after which the vascular endothelial and perivascular cells are invaded by the pathogen, causing increased microvascular permeability due to rickettsial vasculitis [11–14]. Mouse models have shown that rickettsiae are able to cross the blood-brain barrier [15]. One mechanism of bacterial penetration through the blood-brain barrier, besides intercellular penetration of tight junctions and transcellular passage through cells, is through transmigration within infected macrophages [16–18]. It has been suggested that *Rickettsiae*, as other intracellular microorganisms, also might use a transcellular approach, which is supported by the fact that bacterial replication takes place within endothelial cells but the precise mechanisms of rickettsial entry into the CNS have not yet been described [6, 16]. Pathological involvement of the brain is common in fatal cases of rickettsial diseases [19]. Moreover, histological studies have confirmed rickettsial invasion of vascular endothelial cells in the brains of humans [20] and experimentally infected mice [21].

One potential neuropathic role of rickettsiae was suggested in an experimental model showing a significant reduction in survival of rat cerebrocortical neurons as a result of an in vitro infection and intra-cellular decrease in ATP [22]. *Rickettsia rickettsii* has also been shown to infect cerebellar granular neurons from a rodent host, where the infection was shown to induce widespread neuronal degeneration [23]. Another report has demonstrated persistence and reappearance in the CNS, after a relatively long period, of *R. typhi*, in immune-compromised mice developing lethal neurological disorders [24].

Two domestic spotted fever species, *R. helvetica* and *Rickettsia felis*, have been reported in Sweden since 1997 and are considered, with some reservation for *R. felis*, established in the country. Both species have been detected in patients with different types of clinical manifestations. In addition to these, sporadic findings of *Rickettsia monacensis* and *Rickettsia sibirica* have been reported in ticks in Sweden [25, 26]. *R. helvetica* is common in the tick population (*Ixodes ricinus*) in 5–15% of ticks when examining materials from different parts of the country [27]. *R. felis* has the classic dog/cat flea (*Ctenophalides felis*) as a vector, but the vector has not yet been confirmed in Sweden. Most often, a subclinical disease is seen with usually non-specific symptoms and transient fever, which contributes to underestimation of the number of cases, but meningitis, perimyocarditis and facial paresis have also been reported [27–30].

The underlying rationale for the present study was to examine *R. helvetica* survival, growth characteristics, and its influence on cultured NSC-34 mouse motor neurons, that is widely used as an experimental model in studies of motor neuron diseases [31], as well as whether different growth conditions affect the course of the infection. The mouse neuron cell line NSC-34 was in the trials inoculated with a specified quantity *R. helvetica* bacteria and the course of the infection was followed by qPCR, immunofluorescence, confocal microscopy, TEM, TNFα assay and Western Blot.

## Results

### Quantitative real-time PCR (qPCR)

**Fig. 1:**
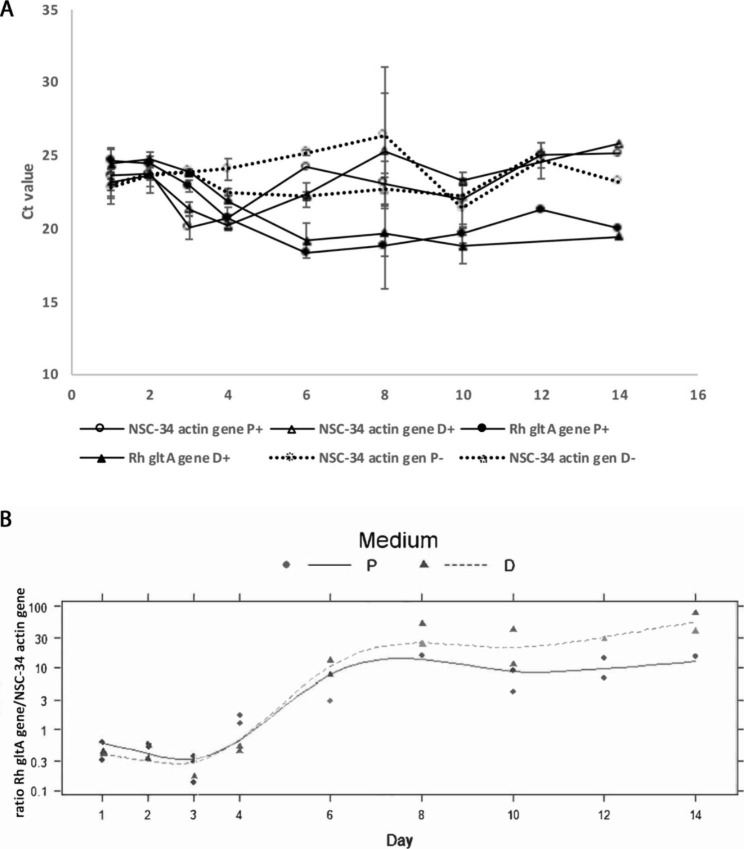
**A-B** Mean values of *R. helvetica* copy number, NSC-34 cells and Rh/NSC-34 ratio (Y-axis) in different media measured by qPCR over 14 days (X-axis). (**A**) The Ct value of the *gltA* gene of *R. helvetica* and the actin gene in proliferation (P) medium (10% FBS) and in differentiation (D) medium (1% FBS). (-) represent uninfected and (+) infected cell medium, respectively. (**B**) The ratio *R.helvetica glta* gene/NSC-34 actin gene shows that the number of *R. helvetica* copies per cell was significantly higher (p < 0.0029) in D-medium from day 6 to 14

After inoculating the NSC-34 cell line with *R. helvetica* bacteria at a ratio of 1:1, i.e. one bacterium per cell in accordance with the calculations based on the standard calibration curves for *R. helvetica* and actin (see below), the number of bacteria was analyzed in relation to the number of cells over 14 days. Samples were taken on days 1, 2, 3, 4, 6, 8, 10, 12 and 14 and a qPCR for *R. helvetica* and mouse actin, the latter as an indirect measure of the number of cells, was run in parallel for each day and on each sample material. The growth experiments were run in duplicat and in both normal proliferation (P) and nutrient-reduced differentiation (D) medium. Figure 1A illustrates by PCR the growth of *R. helvetica* in the NSC-34 cells in P and D medium (Rh P+/ Rh D+). The results represent the mean values of two attempts for each medium for 1–14 days. Figure 1A also shows the number of infected and uninfected NSC-34 cells during the same time measured by PCR against the mouse actin gene. The number of cells, both infected and uninfected, is relatively stable during 14 days of cultivation. In Fig. 1B, the ratio between the number of copies of the *R. helvetica* gltA gene and the NSC-34 cell actin gene is calculated. A significant increase in growth is seen when comparing day 6–14 with day 2–4 (p < 0.001). Growth increases until day 6–8 and then levels off. In addition, there is a significant interaction between day and medium, i.e., the time effect differs between medium with growth in D medium being higher than in P medium (p = 0.0029).

### Immunofluorescence and phalloidin staining of cytoskeleton

**Fig. 2:**
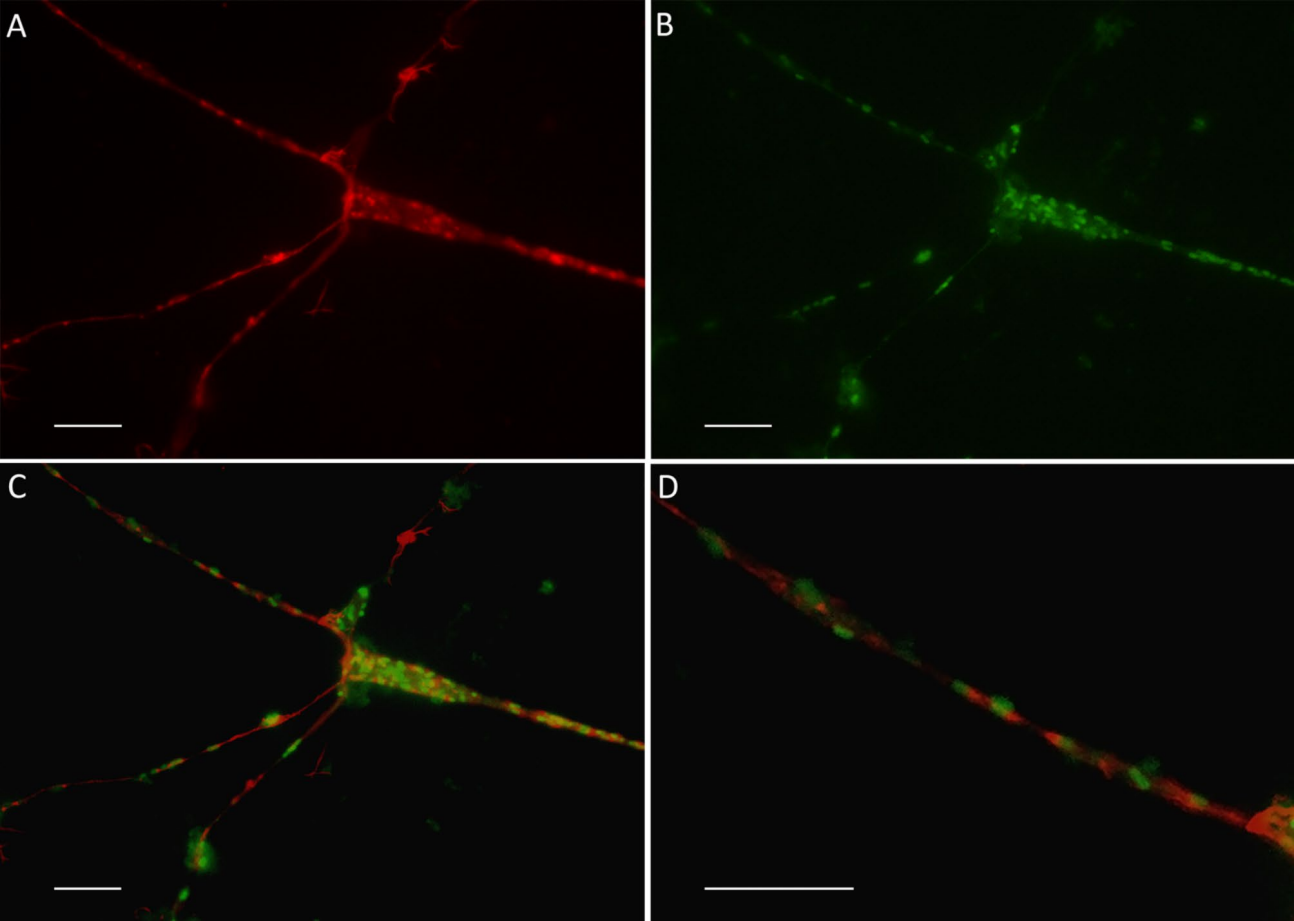
Photomicrograph of *R. helvetica*-infected NSC-34 cells after 12 days of infection in nutrient-reduced D-medium. (A-C original magnification x 1000.) F-actin was stained with phalloidin (**A**), and presence of *R. helvetica* visualized by anti-rickettsial rabbit antiserum and FITC-labelled anti-rabbit immunoglobulin (**B**). In the merged image (**C**), the position of the green, fluorescent FITC-labeled bacteria can be seen next to small red stained actin nodes within the actin network and with close-up view (magnified by 120%) in image (**D**). Result is shown for one representative experiment, performed five times. Bars Fig. 2A-D denote 5 μm

Cells were grown on round glass slides in D-medium and the NSC-34 cells were infected with a known amount of *R. helvetica*, in a ratio corresponding to one bacterium per cell, to study their ability to polymerize actin. In Fig. 2, the bacteria in the mouse neuron cell line NSC-34 appear in green (Fig. 2A) through use of an anti-*R. helvetica* rabbit serum, followed by an anti-rabbit FITC-labelled antibody. The actin network appears in red after using phalloidin labelling (Fig. 2B). In the merged image, the green, fluorescent labelled (FITC) bacteria can be seen within the red actin network (Fig. 2C) and in a close-up picture, Fig. 2D, red actin nodes can be seen next to the green, fluorescent bacteria.

**Fig. 3:**
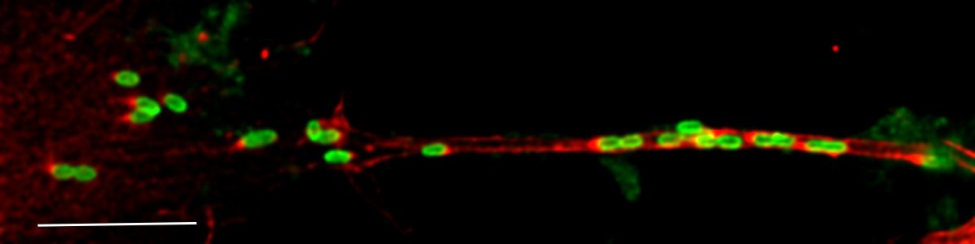
Confocal microscopy of NSC-34 cells in D-medium infected with *R. helvetica* for 6 days. The cells are stained with an anti-*Rickettsia* antibody, followed by an anti-rabbit FITC-labelled antibody (green) and Alexa 568 phallodin (red). Note the red flames at one polar end of the bacterium as an expression of its actin polymerizing activity. Bar denote 5 μm

To increase the resolution and clarify the bacterium’s relationship to the actin network, the infected neuron cultures were also examined in a confocal microscope. It was then clearly seen how the bacteria follow the actin threads and obviously actively polymerize the actin, which appears as red flames at one polar end of the bacterium (Fig. 3).

### Transmission electron microscopy (TEM)

**Fig. 4:**
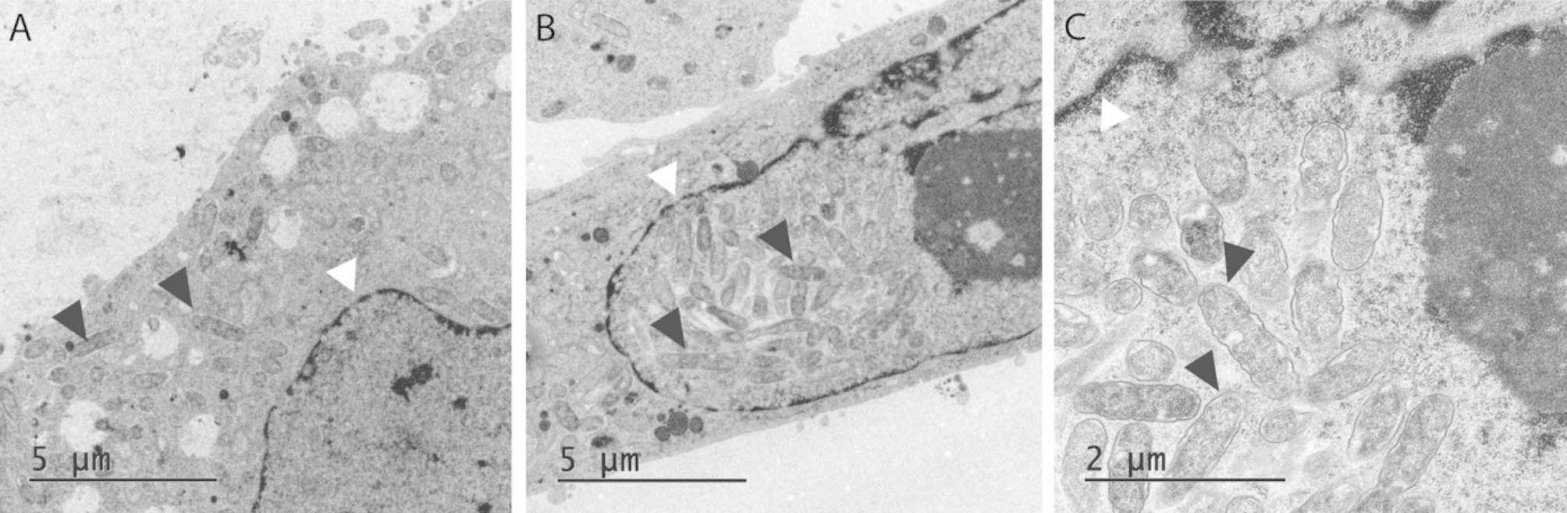
TEM of *R. helvetica*-infected NSC-34 cells in D-medium. An increasing number of rickettsia bacteria were seen with characteristic double membrane (black arrowheads) at Day 8 within the cytoplasm (**A**) and within the nucleus on Day 10 (**B** and **C**). White arrowheads point at the nuclear envelope. Original magnification, x 4200 (**A-B**), and x 11,500 (**C**)

Transmission electron microscopy was performed on NSC-34 cells grown in D-medium in 12-well plates and infected with *R. helvetica* and then transferred to a grid. The characteristic double-membrane rickettsia-bacteria could be detected both in the cytoplasm of most of the cells, as shown in Fig. 4, on Day 8 (A) and 10 (B), and in a few cells, also demonstrated in the nucleus (C), on Day 10, where single up to several bacteria that had invaded the nucleus were seen. In Fig. 4, the nuclear envelope is marked with white arrowheads and bacteria with a black arrowhead.

### ELISA based TNFα assay

**Fig. 5:**
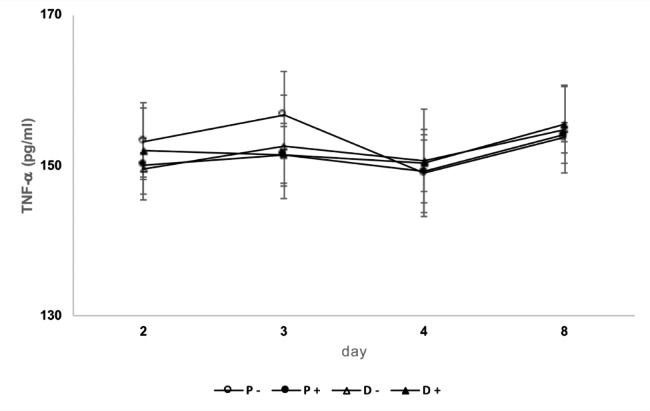
Mean values of TNFα titer concentrations in two cell growth media. The two media consisted of proliferation (P) and nutrient-reduced differentiation medium (D) containing both uninfected (-) and infected (+) NSC-34 cells, during Day 1–8 after infection with *R. helvetica*. The concentration of TNFα was calculated using the standard curve. Experiments with P-medium (10% FBS) were performed twice in duplicates, and with D-medium (1.0% FBS) three times in duplicates. No significant rise in titer or significant difference in titer was seen between infected or uninfected cell media (p = 0.2014 (medium P) and p = 0.7619 (medium D). Black filled circles (P+) and triangles (D+) represent infected cell media and unfilled symbols uninfected media (P-/D-).

NSC-34 cells that were grown for 8 days in either normal proliferation (P) or nutrient-reduced differentiation (D) cell medium and subsequently either infected with *R. helvetica*, or not infected, respectively, were analyzed for their cytokine TNFα content. The experiments were performed twice in duplicate before compilation. No significant rise or difference in TNFα titer could be detected over the course of infection, independent of the cell medium (p = 0.2014 (medium P) and p = 0.7619 (medium D) (Fig. 5).

### Western blot (WB) analysis

The protein expression of the infection differs over time as shown by WB. NSC-34 cells were cultured in P- and D-medium and both *R. helvetica* infected and uninfected cells were checked at several time points on Day 2, 4, 6, 8, 10 and 14, and analyzed using WB with an anti-*R. helvetica* IgG-antibody followed by an anti-rabbit Alexa fluor 555 antibody (Cy3).

**Fig. 6:**
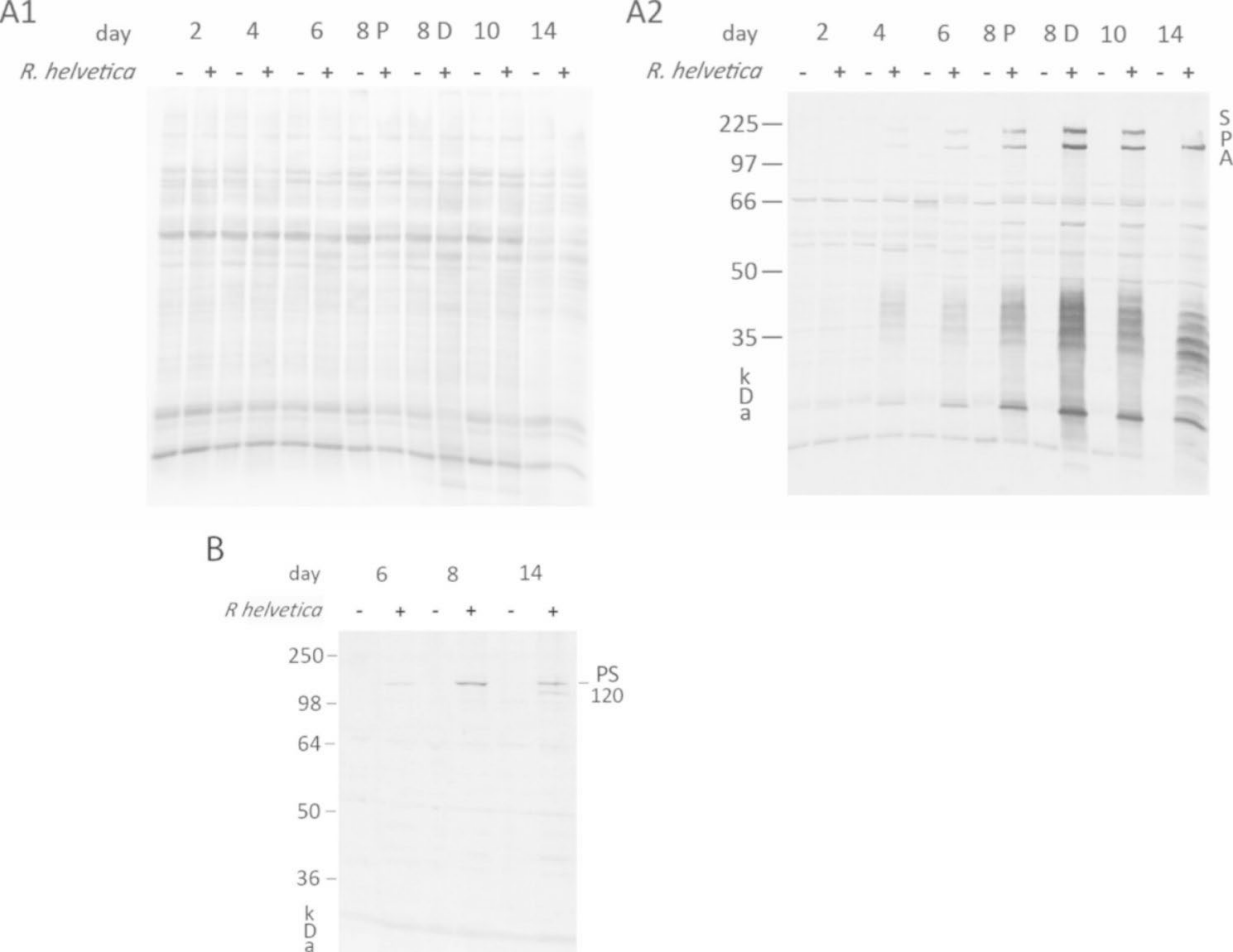
Protein and WB gel on *R. helvetica* infected and uninfected NSC-34 cells, respectively. Gels (A1) and (A2) represent the same whole gel where (A1) shows Cy5-labelled total protein expression showing the total distribution of protein in infected (+) and uninfected (-) NSC-34 cells during a 14-day period. For the timepoints illustrated in the figures the cells were grown in P-medium, except for Day 8 when cells are compared after growing in P- and D-medium respectively and (A2) WB analysis of the same membrane showing rickettsia proteins of *R. helvetica* infected (+) and uninfected (-) NSC-34 cells after 2 to 14 days using an anti-*R. helvetica* IgG antibody labeling of antigenic structures of the rickettsia bacteria. The expressed proteins increase over time and shows the same pattern in both P and D medium but were expressed in greater amount in D-medium which is illustrated Day 8 with both P- and D-medium in comparison. As seen after Day 10, one of the SPAs in size in the 97–225 kDa region was downregulated. (B) Represents six lanes of a Western blot membrane, where the specific anti-Sca4 protein antibody shows that protein expression increases in the infected (+) cells with time and that the downregulated *R. helvetica* protein in A2, after day 10, does not correspond to PS120 which remains expressed. Every other lane represents uninfected (-) NSC-34 cells. The original gels for A2 and B are included as Supplementary files

In Fig. 6A1 and A2, the amount of expressed proteins seen in infected NSC-34 cells increases over time, in concordance with the PCR results of increasing numbers of bacteria in Fig. 1. The total amount of proteins expressed in P-medium (Fig. 6A1) in uninfected (-) and infected (+) cells respectively as well as the specific proteins to *R. helvetica* (Fig. 6A2) showed the same pattern in both P and D medium but were expressed in greater amount in D-medium which is illustrated Day 8 with both P- and D-medium in comparison. That a greater amount of rickettsial proteins can be seen expressed in infected cells, grown in D medium, once again confirms the higher growth in this medium, which was previously also seen with PCR (Fig. 1). Figure 6A2 also demonstrates that the uninfected cells (-) do not express the *R. helvetica* specific proteins. After Day 10, it was also found that expression of one of the surface protein antigens (SPA) in size in the 97–225 kDa region was downregulated. Figure 6A1 shows that the total amount of protein (cells plus bacteria) is similar in infected and uninfected cells over time. Using a primary anti-sca4 protein antibody demonstrated that it was not the Sca4 protein PS120 that was downregulated, but instead remained expressed (Fig. 6B). The original gels for A2 and B are included as Supplementary files (Fig SA2 and Fig SB).

## Discussion

The present study shows that *R. helvetica* infects, survives, and multiplies in NSC-34 cells, a mouse neuron cell line, as shown by PCR, immunofluorescence, confocal microscopy, and TEM, and that, using the latter method, the bacteria were also found invading the cell nucleus. Moreover, it was obvious that the rate of bacterial growth relative to the number of cells was affected by the nutrient content of the cell medium with higher infection rates at lower nutrient content and with differentiated cells. Infected NSC-34 cells were found not to induce TNFα, and the cytokine levels were the same for infected and uninfected cells and independent of cell medium.

The findings also provide support for the presence of actin-based motility, and it could be shown that the expression of protein in infected NSC-34 cells was consistent with the detected number of bacteria in infected cells and that the bacteria did not downregulate the expression of the Sca4 protein PS120, a protein that promotes spread through the host cell membrane. The growth curves and development of the rickettsial load in infected NSC-34 cells were similar to what has previously been observed in infected Vero and L929 cells and human monocytes, but with a higher average of bacteria for nutrient-reduced D-medium and differentiated cells compared to normal proliferation medium [14, 32] (Fig. 1). After one week, a stationary phase in growth was achieved, which was then maintained during the observation period. As an observation, and using the microscope area calculator, it was also noted that the area of FITC-labeled infected and often clustered cells was on average 3 times larger when the cells were grown in D-medium, and *R. helvetica* also appeared to grow and spread more rapidly to adjacent cells under these conditions and in both cases with the same number of bacteria in the inoculum. The reasons for this are not known, but a possible assumption is that the cell’s mechanisms for intracellular killing or inhibition of rickettsial growth, in addition to the effects of the infection itself, are also negatively affected by nutrient-reduced conditions [33, 34]. No significant increase in TNFα levels could be seen in the infected neurons compared to uninfected ones (Fig. 5). This is of interest, as it has been reported that activation induced by TNFα protects the host cell against infection-induced apoptotic death and, thus, is critical for host cell survival but even with the low levels measured during the study, no clear signs of increased cell death were seen [35, 36]. The significance of the lack of TNFα activation is therefore unclear and requires further studies. However, the findings of growth of *R. helvetica* in the cell nucleus of the neuron may indicate that the neuron is damaged by or may even die from the infection. A possible negative impact of the infection is supported by other studies showing that survival of the rat neurons decreased significantly after infection with spotted fever rickettsiae. In that study, the intracellular level of ATP was measured, which was found to gradually decrease and was inversely correlated with the load of rickettsiae, the largest decrease being in the amount of ATP in infection with *R. helvetica* (45%) compared to *R. akari* (31.5%), unlike *R. slovaca*, where no compromised viability of neurons was seen [22]. These findings, together with the current findings indicating neurotoxicity, need to be studied more closely, also with human neurons, to determine whether *R. helvetica* causes cell-damaging effects in neurological or neurodegenerative disease. The demonstrated effects on the mouse neuron of a *R. helvetica* infection, differ to some extent from previously reported findings in infection of human monocytes with *R. helvetica*, where TNFα production was induced and the rickettsia bacteria both replicated and survived in the monocyte’s cell plasma without causing apoptosis of the host cell, which instead theoretically enables the infection to also spread to the CNS by crossing the blood-brain barrier [6, 14]. In the case of *R. rickettsii*, it has also been shown that the bacterium can modulate the proteome in its host cell, tick embryonic cells (BME26), including proteins inscribed with apoptosis, and exert an inhibitory effect on the apoptosis, which helps ensure its proliferation in that cell [37]. The spotted fever group of rickettsiae, in contrast to most of the typhus group, have the capacity to move in an intra- and intercellular fashion that involves protrusion formation, engulfment and vacuolar escape [38, 39]. Previous studies have given indications that *R. helvetica* invade nuclei [14, 32] while others have suggested the opposite, namely that *R*. *helvetica* lacks intracellular motility, which makes it unlikely that nuclei will be invaded [40]. It was obvious, however, that the bacteria could be detected in the cell nucleus of single mouse neurons, and the TEM images demonstrated not just one but several bacteria inside the cell nucleus (Fig. 4C). The process of entering the nucleus has been suggested to be an active one, driven by actin polymerization, but we did not capture any bacteria in the image, bound to actin tails and about to penetrate the cell nucleus [38]. Actin-based motility is important though it contributes to cell-to-cell spread and dissemination of bacteria within the host. The infected NSC-34 cells developed filopodia and the actin cytoskeleton, ranged from short to longer tails or branched structures. When staining the cytoskeleton of the NSC-34 cells with phalloidin, small red nodes were seen on the skeleton (Fig. 2D). When also staining the bacteria with FITC-labelled antibody, these polar nodes or rather red flames are seen located directly next to and at one polar end of the bacteria, which is particularly evident in confocal microscopy (Figs. 2D and 3). Our interpretation of these nodes or flames is that the bacteria polymerize the cells actin, which means that they probably can move shorter distances intracellularly. *Rickettsia* has genes encoding two different actin assembly proteins: RickA and Sca2. The RickA gene is present in SFG species but is absent in TG species [41].

Ultrastructural studies of the rickettsial actin tail in Vero cells has revealed that the typhus group rickettsiae, *Rickettsia prowazekii* and *Rickettsia typhi*, to have no actin tails and short (1 to 3 μm) straight or hooked actin tails, respectively unlike *R. rickettsii* who has long (> 10 μm) tails that are frequently comprised of multiple, twisting, actin bundles [42]. The motility of the SFG species *R. parkeri* is shown to be different at different times during the infection. Early after invasion, motility is slow and meandering, generating short, curved actin tails. Later in infection, motility is faster and directionally persistent, resulting in long, straight actin tails [43]. In the case of *R. parkeri* it has also been shown that actin-based motility may be necessary to be able to move to the host cell membrane but may not be needed to initiate or prolong protrusions [44]. The Sca4 surface cell protein has recently been identified as another protein from SFG rickettsia that specifically promotes spread by protrusion engulfment and is thought to be secreted from the bacteria [44]. It has been reported that Sca4 from *R. parkeri* binds to the cell adhesion protein vinculin and inhibits its activity, thereby reducing intercellular tension at cell-cell junctions, which promotes and enables proliferation [44, 45].

We followed whether the protein expression of bacteria differed over time when culturing *R. helvetica* in NSC-34 cells for 14 days (Fig. 6). It turned out that expression of the amount of protein increased over time and began to stabilize starting on about Day 8, which corresponded to the bacterial growth measured using qPCR. We also saw, after Day 10, that expression of one of the surface protein antigens (SPA) in size in the 97–225 kDa region was downregulated. Using an anti-Sca4 protein antibody in WB, it became apparent that the downregulated protein band did not correspond to the Sca4 protein PS120, which is thus expressed. It seems likely that the expression of Sca4 protein remains when bacterial growth and spread between cells takes place in the cell culture.

To better understand the mechanisms underlying formation of actin polymerization and actin-based motility, and factors for protrusion as well as the different genes and encoded proteins that are crucial for these processes and what it means for the adaptation and pathogenicity of the *R. helvetica* bacterium, further studies are needed applying molecular biological and proteomic analysis tools, for comparison of data in different phases of the infection cycle.

## Conclusions

Taken together, our data suggest that *R. helvetica* polymerizes host cell actin, which means that they can move shorter distances intracellularly. Furthermore, there are indications that the infection could be harmful to NSC-34 mouse neurons because some cell nuclei were invaded by bacteria and no increase in TNFα production was seen, which - together with data from previous studies showing that the neurons’ ATP production is strongly inhibited - points in the same direction. This may suggest that *R. helvetica* may be harmful to the neuron cell, under these in vitro conditions, but the full effects of the infection on the cell need to be studied further, also on human neurons, to also understand the possible significance of this infection in relation to pathogenetic mechanisms.

## Materials and methods

### Cell culture conditions

Vero cells (ATCC, Manassas, VA, USA) were grown in cell monolayers in TC-75 culture flasks in complete Dulbecco’s modified Eagle medium: nutrient mixture F-12 (DMEM F-12) (Thermo Fisher Scientific,), 1% penicillin/streptomycin (Thermo Fisher Scientific), 1% glutamine (Thermo Fisher Scientific) and 10% fetal bovine serum, (FBS) (Sigma-Aldrich) at 37 °C and 5% CO_2_ as previously described [32, 46].

The mouse motor neuron cell line NSC-34 cells (Cat. no. CLU140: Cedarlane, Burlington, Canada) is a hybrid cell line, produced by fusion of motor neuron enriched, embryonic mouse spinal cord cells with mouse neuroblastoma. The cell line contains two populations of cells: small, undifferentiated cells that have the capacity to undergo cell division and larger, multi-nucleate cells. Both cells express many properties of motor neurons and were grown in a proliferation medium, hereafter referred to as P-medium containing DMEM (Sigma-Aldrich) without sodium pyruvate with 1% penicillin/streptomycin P/S), 1% glutamine and 10% FBS in a humidified atmosphere containing 5% CO2 at 37 °C as previously described [47]. Cultures underwent 5–10 passages. Next, the NSC-34 cells were cultured both in a P-medium and in a nutrient-reduced medium with a lower content of FBS (1.0%), 1% P/S, 1% Glutamine and Dulbecco’s modified Eagle medium to enhance differentiation (D-medium) [31]. In the latter case, some of the cells died but the remaining differentiated cells were allowed to grow and develop long neurites. Both kinds of medium were changed regularly every two to three days to fresh medium. In addition to the differentiated cells, the cells in P medium were also used for comparison in some of the further experiments. Before inoculation with *R. helvetica*, cells were seeded in a 12-wells plate at a concentration of 100,000 cells/well with 4 mg/ml Cotrimoxazole (Sigma-Aldrich) instead of penicillin/streptomycin so as not to inhibit the growth of *R. helvetica*.

### Rickettsial strain and inoculation in Vero and NSC-34 cell lines

An isolate of *R. helvetica*, genetically characterized by PCR and genomic sequencing, from an *Ixodes ricinus* tick previously propagated in Vero cells plated in TC-75 culture flasks, and harvested as before described, was used [48, 49]. Uninfected Vero cells were then infected with frozen ampoules of a suspension of these previously infected Vero cells and, as previously recommended, centrifuged at 1000 x g for 30 min to facilitate contact between the bacteria and the cells [50]. The cell cultures were then incubated in a humid cell chamber in 5% CO_2_, 32 °C. On the post-infection Day 6, the *R. helvetica* bacteria were purified by first detaching the cells with trypsin-EDTA (Thermo Fisher Scientific), washed in ultrapure H_2_O two times, sonicated for 6 × 5-second pulses, with 10-second pauses at 40% amplitude, and finally centrifuged at 1000 x g for 10 min to remove the cell content. Using quantitative real time PCR (qPCR), the supernatant was analyzed for *R. helvetica* with probe and primers targeting the citrate synthase-encoding gene (*glt*A), as described previously [51]. The number of copies was determined using a standard calibration curve as described below.

The NSC-34 cells were then inoculated with a known amount of *R. helvetica* bacteria at a 1:1 ratio, i.e. 100,000 cells and 100,000 bacteria/well, and the cells were centrifuged for 10 min at 1000 x g (Sigma Qiagen 4K15 Centrifuge) and then cultured for 1, 2, 3, 4, 6, 8, 10,12 and 14 days in a humidified atmosphere containing 5% CO2 at 32 °C, in both the P-medium and D-medium, and in both cases with the addition of 4 ug / ml Cotrimoxazole. Correspondingly and during the same period, uninfected NSC-34 cells were cultured in P-, and D- medium, respectively.

### qPCR

Vero and NSC-34 **c**ells were washed twice with PBS before being scraped off the bottom of the wells with a spatula and moved to Eppendorf tubes in 200 µl PBS. Total cellular DNA was isolated on days 1, 2, 3, 4, 6, 8, 10, 12 and 14 from both infected cells and non-infected controls using QIAamp DNA mini kit protocol according to the manufacturer’s instructions (Qiagen AB). For quantification of DNA copies, a genus-specific realtime qPCR was assayed as above in a Rotor-Gene 3000 (Corbett Research Ltd) using LightCycler® TaqMan® Master (Roche Diagnostics) [51]. To analyze the bacteria-to-cell ratio, a qPCR for mouse actin was also performed using the primers fwd 5‘- TAG GCA CCA GGT AAG TGA CC and rev 5‘ - ACC CAT TCC CAC CAT CAC AC with the probe 5’ 6FAM-CGG TGC TAA GAA GGC TGT TCC CTT CCA CA–BBQ and the LC Taqman Master kit (Roche Diagnostics). Annealing temperature was 58 °C, elongation time was 25 s and cycle repeat was 45 times. (95 °C 15s, 58 °C 15s, 72 °C 25 s) x 45. The number of *R. helvetica* bacteria was calculated using a standard calibration curve for *R. helvetica* plasmid pCR4-TOPO cloning vector containing 1.5 to 1.5 × 10^8^ copies of the cloned *glt*A fragment (nt1126-1199) of *R. helvetica*, diluted in 10-fold series between 20 and 20 000 copies [32, 51]. For mouse actin, the standard curve was prepared from diluting a known concentration of cells that was determined by counting the cells using a Bürker chamber.

### Immunofluorescence and staining of cytoskeleton actin by phalloidin

NSC-34 cells were seeded on round glass covers in 12-well plates at a concentration of 100,000 cells/ml and in differentiation D-medium (1.0% FBS). They were infected with an inoculum corresponding to 100,000 *R. helvetica* bacteria/well, as described above, in a cell:bacteria ratio of 1:1 and checked daily post infection by immunofluorescence. After 10 days of incubation, when the cells had grown and differentiated, the wells were washed carefully one time with PBS and then incubated with 4% paraformaldehyde in PBS for 10 min at room temperature. The wells were then washed twice with PBS before incubation with 0.1% triton X-100 in PBS for 5 min at room temperature. After two washes with 0.1% triton X-100 in PBS, the round cover glasses were picked up and glued to a glass slide with transparent nail polish. The cells were stained with a rabbit anti-*R. helvetica* antibody (1:1000) [52] that was added to the round cover glass and then incubated for 1 h at room temperature. After two washes with 0.1% triton X-100 in PBS, an anti-rabbit fluorescein antibody (1:80, Cat. no. 5230 − 0298, Seracare, USA) was added to the cells and incubated for another hour at room temperature. The same wash was repeated before incubation with phalloidin (1:40, Cat. no. A12380; Thermofisher Scientific,) for 10 min at room temperature. One wash with 0.1% triton X-100 and then the last wash in PBS before mounting the cover glass with fluor shield containing DAPI (VWR, International AB). The glasses were inspected in a Nikon Eclipse E600 light microscope at x400 and x1000 magnifications, and the findings were documented using an NIS-Elements BR 5.30.01 camera system.

### Confocal microscopy

On Day 6, when the growth of bacteria in the cells had increased significantly in accordance with the PCR findings, the cells were seeded and stained as described above. Confocal images were obtained with a Zeiss LSM 700 confocal microscope equipped with 405, 488 and 555 nm lasers, with emission range 420–550, < 606 and > 568 respectively. A 63x NA.14 oil objective was used, pixels 40 nm, Z series with 130 nm steps and the ZEN (black edition) Software. Images were deconvolved with Huygens Professional software (Scientific Volume Imaging) before analyzed using the open-source Java application ImageJ (https://imagej.nih.gov/ij/).

### TEM

On Day 8 and 10 post-infections with *R. helvetica* in nutrient-reduced D-medium (1.0% FBS), as mentioned above, NSC-34 cells were washed with PBS and then fixing solution was added. The cells were then prepared for transmission electron microscopy in the following way. Fixation and embedding: Cells were fixed in 2.5% Glutaraldehyde (Ted Pella Inc) and 1% Paraformaldehyde (Sigma-Merck Sweden AB,) in 0.1 M Phosphate buffer (PB) pH 7.4. Cells were rinsed in 0.1 M PB for 10 min prior to 30 min incubation in 1% Osmium tetroxide (TAAB, Aldermaston England) in 0.1 M PB. After an additional rinse in 0.1 M PB, cells were dehydrated using increasing concentrations of ethanol (50%, 70%, 95% and 99.9%) for 10 min each step. The wells were then filled with a mixture of Eponate 12™ resin kit (Ted Pella Inc,) and 99.9% EtOH (1:1) for 1 h, followed by 100% resin, and left-over night. The next day, the resin was replaced with freshly prepared resin and then polymerized at 60 °C for 48 h. Sectioning and contrasting: Ultrathin Sects. (60–70 nm) of the cell layer were made in an EM UC7 Ultramicrotome (Leica) and placed on grids. The sections were subsequently contrasted with 5% uranyl acetate and Reynold’s lead citrate and visualized using an Tecnai™ G2 Spirit BioTwin transmission electron microscope (Thermo Fisher/FEI) with an ORIUS SC200 CCD camera and Gatan Digital Micrograph software, both from Gatan Inc., Pleasanton CA, USA.

### Cytokine measurements

Cells were grown in D- and P-medium and infected as described earlier. The cell medium from infected and uninfected NSC-34 cells grown for 2, 3, 4 and 8 days were collected, and ELISA analysis was performed to measure the levels of mouse neuron TNFα using a standard ELISA kit (Invitrogen, ThermoFisher Scientific) and following manufacturer’s protocols. Briefly, standards and samples were added together with the biotin conjugate to plates already preincubated with antibodies and blocked. After two hours of incubation and a wash step, a detection antibody was applied, and after complete incubation and another wash step, the substrate was added. Color development was monitored after approximately 10 min at 450 nm using a VersaMax plate reader (Molecular devices, San Jose). To generate the standard curve, cell culture medium spiked with TNF-α were titrated to obtain eight different concentrations spanning a broad range from 0 to 2,000 pg/ml. All eight samples were assayed simultaneously to obtain their corresponding fluorescent signals (450 nm) according to the manufacturer’s instructions.

### Western blot

NSC-34 cells were grown and infected as described above, using P-medium (10.0% FBS) but also D-medium (1.0 FBS) which was analyzed for comparison. After 2, 4, 6, 8, 10 and 14 days, the cells were washed twice with PBS before being scraped off the wells with a spatula in 100 µl PBS. The cells were centrifuged for 10 min at 13,000 rpm, and the pellet was dissolved in 100 µl labelling buffer (Cytiva Sweden AB). After a 3-minute incubation period at 95 °C, the total protein content of the samples was labelled with Cy5 (Cytiva Sweden AB) for 30 min at room temperature. The same volume of 2x sample loading buffer (2.1% SDS, 65, 8 mM Tris-HCl pH 6.8, 26.3% glycerol (w/v), 0.01% bromphenol blue) with 40 mM DTT (Invitrogen,ThermoScientific) was added, and the samples were boiled for another 3 min at 95 °C and centrifuged at full speed for 3 min before being loaded onto a 12% Tris-glycine pre-cast gel (Invitrogen, Thermo Fisher Scientific). After electrophoresis, the proteins were transferred to a PVDF membrane (Thermo Fisher Scientific) for 45 min in transfer buffer (25 mM Tris, 192 mM glycine, 10% methanol). The membrane was blocked in 5% bovine serum albumin (BSA) (Sigma-Aldrich) in PBS for 30 min and the primary antibody (rabbit anti-*R. helvetica* antibody 1:2500) was added after one-minute washes, repeated twice, of the membrane in PBS-T (PBS with 0.1% Tween 20, Bio-rad laboratories). After incubating with the primary antibody for 1.5 h at room temperature, the membrane was washed three times in PBS-T before adding the secondary antibody (anti-rabbit Alexa Fluor 555 1:2500, Life Technologies, Thermo Fisher Scientific) for 1 h at room temperature. The membrane was washed three times in PBS-T, dried and scanned at the two wavelengths detecting Cy5 (total protein) and Alexa 555 (immunodetection by antibody) using the Amersham blotting system. An additional primary antibody, a polyclonal anti-sca4 protein antibody, obtained from the Department of Biology, the Massachusetts Institute of Technology, USA was used in a 1:4000 dilution to illustrate the Sca4 protein PS120 [44, 52].

### Statistical analysis

Statistical analyses of data were performed using t-test, ANOVA and a linear (regression) model. R version 3.5.0 statistical software (www.r-project.org) and SAS® proprietary Software 9·4 (TS1M6), licensed to Uppsala University and executed on the X64_10 PRO platform were both used for statistical analysis.

## Electronic supplementary material

Below is the link to the electronic supplementary material.

Supplementary Material 1

Supplementary Material 2

## Data Availability

The datasets analyzed during the current study are available in the [GenBank] repository, [MK189462.1, MK156098.1, AF181036.1, EU407140.1, EU407139.1. Other data supporting the conclusions of this article are included in this published article [and it’s supplementary information files].

## Abbreviations

ATP: Adenosine triphosphate
BSA: Bovine serum albumin
FBS: Fetal bovine serum
DAPI: Diamino-phenylindole
EDTA: Ethylenediaminetetraacetic acid
NSC-34: Mouse motor neuron-like hybrid spinal cord cell line
DTT: Dithiothreitol
PBS: Phosphate buffered saline
qPCR: Quantitative polymerase chain reaction
Sca4: Surface cell protein 4
TEM: Transmission electron microscopy
TNF: Tumor necrosis factor
