# Supplementary figures and images for: Signatures in in vitro infection of NSC-34 mouse neurons and their cell nucleus with Rickettsia helvetica
Source: BMC Microbiol. 2023 Apr 21;23:113. doi: 10.1186/s12866-023-02859-0 (PMC10120103; doi:10.1186/s12866-023-02859-0)

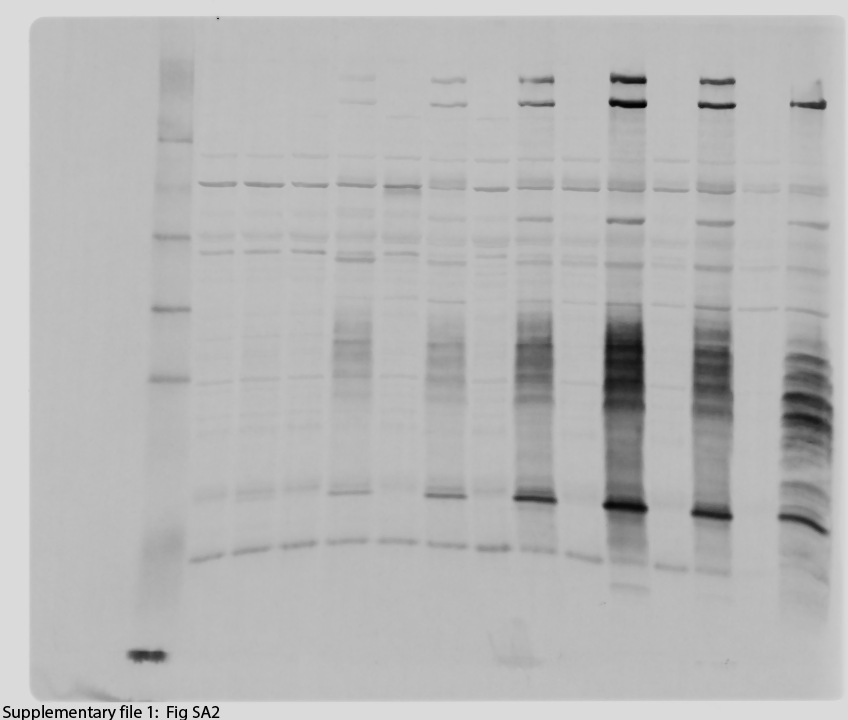

Supplement: Supplementary file 1 — Supplementary Material 1 [file 12866_2023_2859_MOESM1_ESM.png]

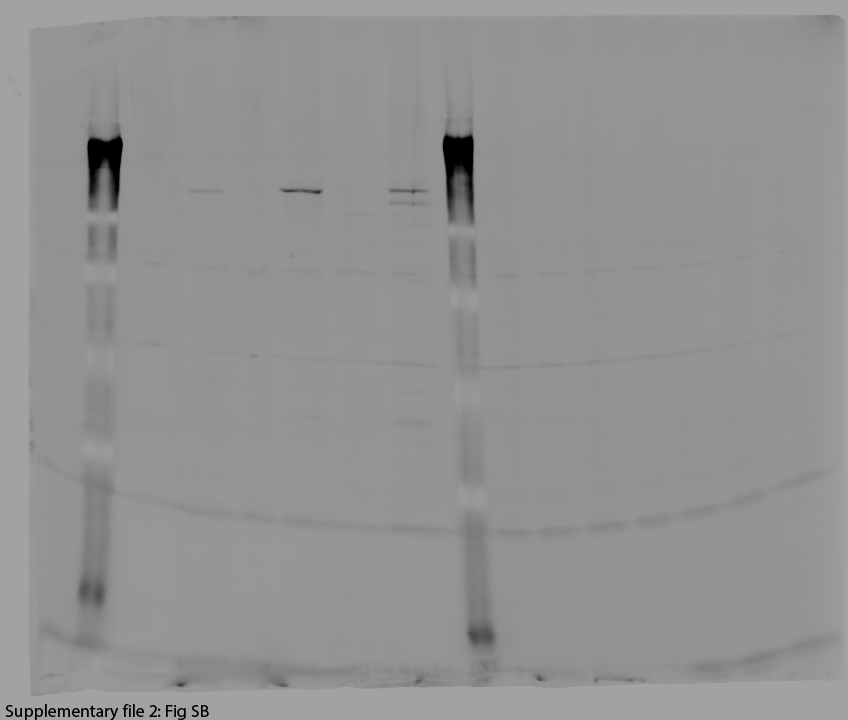

Supplement: Supplementary file 2 — Supplementary Material 2 [file 12866_2023_2859_MOESM2_ESM.png]
